# High-capacity optical long data memory based on enhanced Young’s modulus in nanoplasmonic hybrid glass composites

**DOI:** 10.1038/s41467-018-03589-y

**Published:** 2018-03-22

**Authors:** Qiming Zhang, Zhilin Xia, Yi-Bing Cheng, Min Gu

**Affiliations:** 1 Laboratory of Artificial-Intelligence Nanophotonics and CUDOS, School of Science, Melbourne, VIC 3001 Australia; 2 Department of Materials Science and Engineering, Faculty of Engineering, Monash University, Clayton, VIC 3800 Australia; 3 School of Materials Science and Engineering, Wuhan University of Technology, Wuhan, Hubei 430070 China; 4 State Key Laboratory of Advanced Technology for Materials Synthesis and Processing, Wuhan University of Technology, Wuhan, 430070 Hubei China; 5 Centre for Micro-Photonics, Faculty of Science, Engineering and Technology, Swinburne University of Technology, Hawthorn, VIC 3122 Australia

## Abstract

Emerging as an inevitable outcome of the big data era, long data are the massive amount of data that captures changes in the real world over a long period of time. In this context, recording and reading the data of a few terabytes in a single storage device repeatedly with a century-long unchanged baseline is in high demand. Here, we demonstrate the concept of optical long data memory with nanoplasmonic hybrid glass composites. Through the sintering-free incorporation of nanorods into the earth abundant hybrid glass composite, Young’s modulus is enhanced by one to two orders of magnitude. This discovery, enabling reshaping control of plasmonic nanoparticles of multiple-length allows for continuous multi-level recording and reading with a capacity over 10 terabytes with no appreciable change of the baseline over 600 years, which opens new opportunities for long data memory that affects the past and future.

## Abstract (web summary)

Storing and reading data for long periods of time is of huge interest. Here, the authors demonstrate long optical data memory with nanoplasmonic hybrid glass composites. They show continuous multi-level recording and reading with a capacity over 10 terabytes with no change of the baseline over 600 years.

## Introduction

According to the latest report of the whitepaper from International Data Corporation (IDC) in 2017 (http://www.seagate.com/our-story/data-age-2025/), the information growth rate is much faster than their prediction from 2010 (https://www.emc.com/collateral/analyst-reports/idc-digital-universe-are-you-ready.pdf), and IDC 2012 (http://www.emc.com/leadership/digital-universe/2012iview/). By 2025, the total amount data will reach at more than 160 ZB (10^9^ TB), which is 4 times higher than the prediction made in 2012. The fast growth of big data centers is motivating scientists and engineers to study and record phenomena that last over centuries, which emerges as long data. Long data^1–12^ provides a deep perception of our world by looking at the information in a long time scale. Research in astrophysics, biology, geography, social science, and business generate a large amount of data that has to last over long periods of time to be meaningful. In astronomy, the Square Kilometre Array (SKA) radio telescope produce 576 petabyte (PB) of raw data per hour (https://www.skatelescope.org/news/raeng-grant-to-engage-with-ska-engineering), and the success of in Laser Interferometer Gravitational-Wave Observatory (LIGO) has stimulated the observation of gravitational waves of big astronomic events over century-long^3^. Geologists and ecologists extract long data in temperature and atmosphere levels from ice cores and growth rings in trees to understand the climate changes^4–6^. In life science, long-term experiments have been carried out to study the evolution and mutation of genes^7–10^. Furthermore, the BRAIN initiative in the USA will generate one yottabyte (10^12^ terabyte (TB)) of data to fully understand the human brain. Other fields such as social science and economics are routine now to analyze the information dated back over a long time^11,12^.

To provide long data for the future, the information has to be sampled and kept over generations. Individual storage devices with both high capacity and long lifespan are essential for long data memory. For example, to study the mutation of a human family tree, 8 TBs of data over 500 years are required to analyze the genomes in 10 generations^13^. These stringent requirements constitute an insuperable challenge for existing data storage techniques. Although the ubiquitously used hard disk drives (HDDs) based on the magnetization reversal have approached to their theoretical capacity limitation as high as 2 TB per disk^14^, the reduction of the size of a single information bit leads to a significant degraded stability susceptible to the thermal fluctuation of the environment^15^. Thus, HDDs with high capacity but low lifespans of 2 years^14^ are not suitable for long data memory.

Recently, new data centers based on optical disks have emerged due to the relative longer lifespans compared to HDDs. Panasonic and Hualu Group Co. announced the establishment of a large-scale data center with optical blue ray disks with a total capacity of 1,000 PB^16^. In the meantime, new generation of optical disks announced by Sony and Panasonic^17^ based on low Young’s modulus polymers (~50 MPa^18^) have increased the lifespan from 20 years to 50 years. On the other hand, inorganic ceramic materials with high Young’s modulus have been utilized to demonstrate optical data storage with a lifespan of over 1000 years in commercial products (http://www.mdisc.com/). Recent researches have demonstrated that inorganic bulk materials with high Young’s modulus such as silica glass and diamonds (>50  GPa^19^) have an unequivocally long lifespan^20–22^. Unfortunately, these high Young’s modulus materials have only been able to demonstrate a relatively low capacity, around or less than 100 gigabyte (GB). These optical data memory techniques with long lifespan, but low capacity, in individual disks are not suitable for long data memory. The inorganic materials cannot be combined with versatile functions of nanoparticles, such as gold nanorods^23–25^ for further improving the data capacity due to the huge discrepancy in processing temperatures between bulk inorganic materials and nanoparticles. Consolidation of bulk inorganic materials requires a high-temperature sintering process, which is usually far higher than the melting temperature of most nanoparticles.

Here, we show the development of optical long data memory in a nanoplasmonic hybrid glass matrix. Our result presents an optical recording material that exhibits the data capacity up to tens of TB with a recording and reading time up to 600 years. The up-to-two-order-of-magnitude enhanced local Young’s modulus through the incorporation of an inorganic phase leads to an enhanced thermal stability with an effective capacity of 10 TB per disk. The baseline variation of data over multiple low-capacity optical disks can now be evened by long data memory based on a single optical disk. Such high-capacity and long-lifespan devices allow for continuously recording and reading information with an unchanged baseline for over 600 years in a single disk.

## Results

### Preparation of the nanoplasmonic hybrid glass composites

**Fig. 1:**
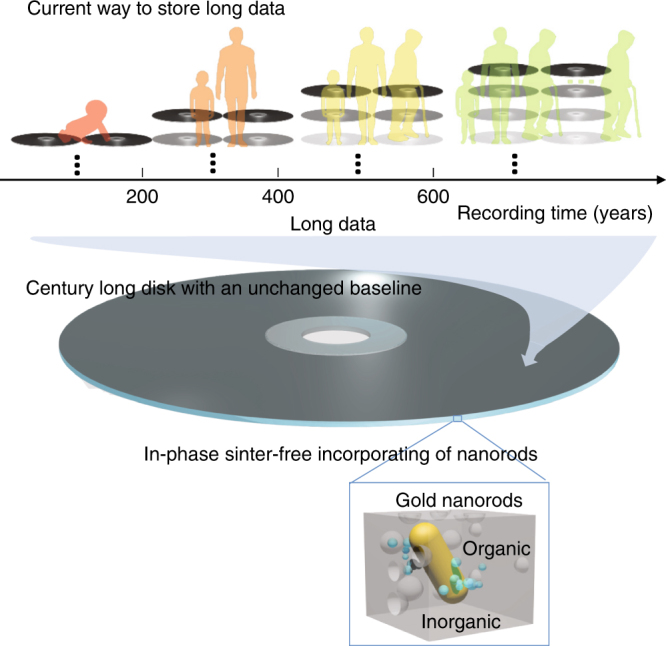
Century-long optical data memory with an unchanged baseline with nanoplasmonic hybrid glass composites. Transformation from the current way to store long data with the variation of baselines into the century-long optical data memory with an unchanged baseline in nanoplasmonic hybrid glass composites. The gray-scale in the disks indicates the change of the baseline over time. Inset: Schematic drawing of the nanoplasmonic hybrid glass composites

To maintain the security and integrity of the long data, the baseline of data, which includes signal contrast and data errors rates, should be unchanged during such a long period of time. Therefore, apart from a long lifespan, the capacity of single disk for long data memory should be high enough to store the whole long data to avoid the variation of the baseline over many disks, as shown in Fig. 1. Optical long data memory can be developed in a nanoplasmonic hybrid glass matrix. The nanoplasmonic hybrid glass composite was formed by a sol–gel process to incorporate gold nanorods into an organic–inorganic hybrid glass composite, often called organic modified ceramic (ORMOCER)^26^, as shown in the inset of Fig. 1.

**Fig. 2:**
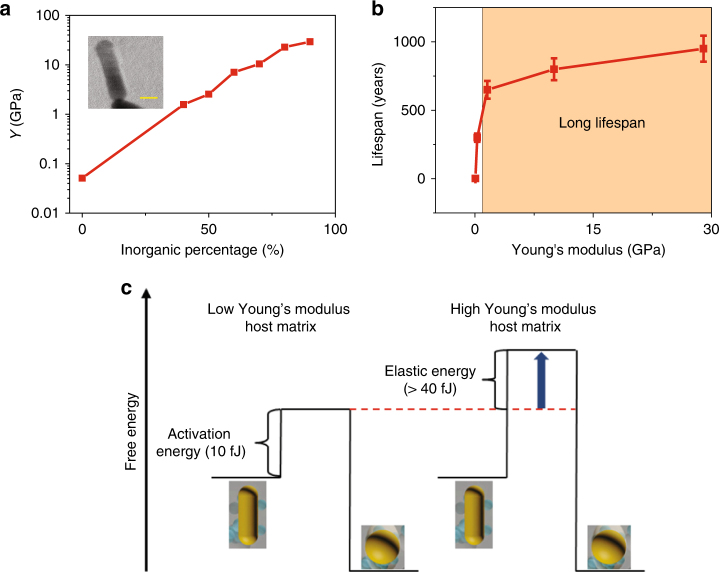
Long lifespan of optical data memory in nanoplasmonic hybrid glass composites. **a** Young’s moduli (*Y*) of the nanoplasmonic hybrid glass with different inorganic percentages. Inset: Transmission electron microscope image of the nanoplasmonic hybrid glass composites. **b** Lifespan of the gold nanorods in the nanoplasmonic hybrid glass composites with different Young’s moduli. Standard deviations are represented by error bars. **c** Simplified effective energy barrier model of the data memory with gold nanorods

The sol–gel process at room temperature allows the sintering-free incorporation of nanorods into the organic–inorganic hybrid glass composites as a host matrix without tampering the shape of the nanoparticle, where the shape transition between rods and spheres can be employed for the information recording. The incorporation of the inorganic phase increases the local Young’s modulus of the host matrix around nanorods to improve the lifespan of the shape of nanorods by removing the unwanted shape degradation susceptible to the environmental thermal perturbation and, hence, an enhanced lifespan of the data memory. To this purpose, gold nanorods were dispersed into the mixture of organic–inorganic hybrid glass composites^26^ consisting of organic (polyethylene glycol (PEG)) and inorganic (silica) components (See Methods and Supplementary Note 1). The mixed solution was then solidified by heating at a temperature of 313 K, which was well below the melting point of gold nanorods. One example of the transmission electron microscope (TEM) images of the samples is shown in the inset of Fig. 2a. The surface of the gold nanorod is uniformly covered with the hybrid glass composites without any gaps.

In Fig. 2a we show the engineered Young’s modulus of the hybrid glass composites through different ratios between the organic and inorganic components (See Methods for the measurement process). As the percentage of the inorganic phase increases, the Young’s modulus of the hybrid glass composites increases due to the very-high chemical bond strength in silica. The highest Young’s modulus of the materials with 90% inorganic components is 29 GPa, which is close to half of that of the fused silica (72.4 GPa)^19^. Thus we expect the lifespan of the host matrix at a high inorganic level is close to the fused silica glass. To analyze the lifespan of the optical data memory, a simplified effective energy barrier model of the data storage with gold nanorods is shown in Fig. 2c. The data are stored in the energy minima of the shapes of the gold nanoparticles between the rods and sphere shapes. According to the theoretical simulation^27^, gold nanorods have a free energy higher than that of the spheres. Thus the lifespan of the optical data memory with nanoplasmonic hybrid glass composites mainly depends on the lifespan of the shape of nanorods. To achieve a shape change in the new material, additional activation energy of gold nanorods is essential for the shape transition of the nanorods to overcome the elastic energy associated to the enhanced Young’s modulus during the expansion of the host matrix (see Supplementary Note 2). Thus, an increase of the effective activation energy drastically enhances the lifespan of the shape of gold nanorods according to the Arrhenius Law^28^.

### Long data memory in nanoplasmonic hybrid glass composites

The significantly enhanced lifespan of the shape of gold nanorods was corroborated in the accelerated aging experiment. According to the Arrhenius law^28^, the lifespan (*L*) of any degradation process at a given temperature (*T*) can be evaluated by

1 $$L = A\exp (E_a/k_BT)$$

where *E*_a_ is the activation energy of the degradation process, *A* is the pre-exponential factor, *T* is the absolute temperature, and *k*_B_ is the Boltzmann constant. Thus, heating the sample to an elevated temperature is equivalent to age the sample for a long time at room temperature. *L* at high temperature was acquired when the intensity of the fluorescent signal of the gold nanorods decreases to a half of the value before heating.

By fitting the acquired L at high temperature with eq (1) (Supplementary Note 3), *L* at room temperature (300 K) of the hybrid glass composites with different ratios between the organic and inorganic components is shown in Fig. 2b. Indeed, the lifespan of the shape of gold nanorods increases as the ratio of the inorganic component increases. The influence of humidity on the lifespan was also studied (Supplementary Note 3). A lifespan longer than 650 years at room temperature can be achieved when the local Young’s modulus is enhanced to 1.57 GPa, which is highly desired for the long data storage, such as the mutation of genes of a family tree^13^.

**Fig. 3:**
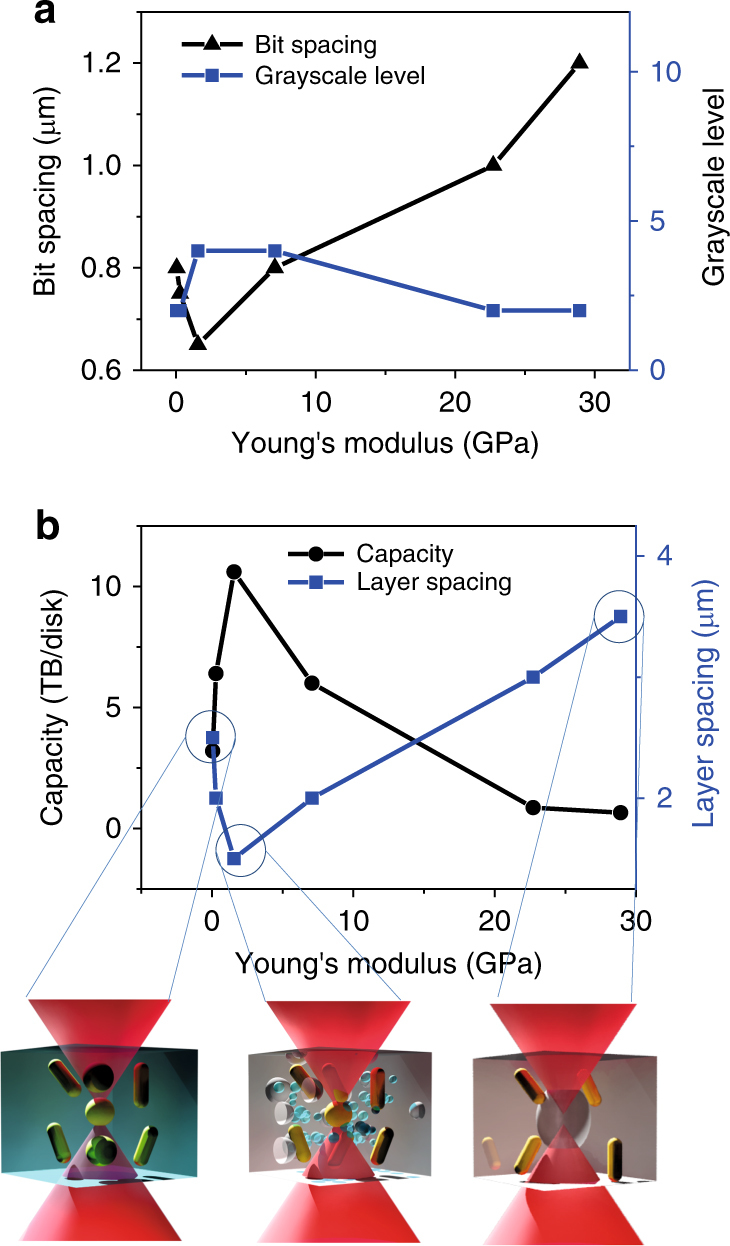
High-capacity optical data memory in nanoplasmonic hybrid glass composites. **a** Bit spacing and gray-scale level of optical data memory of nanoplasmonic hybrid glass composites with different Young’s moduli. **b** Capacity and layer spacing of the optical data memory in nanoplasmonic hybrid glass composites. Inset: Schmatic drawing of laser melting of gold nanoparticles in low Young’s modulus (Left), laser melting of gold nanoparticles with smaller spot size in enhanced Young’s modulus (Middle), and laser induced micro-explosion in high Young’s modulus (Right)

**Fig. 4:**
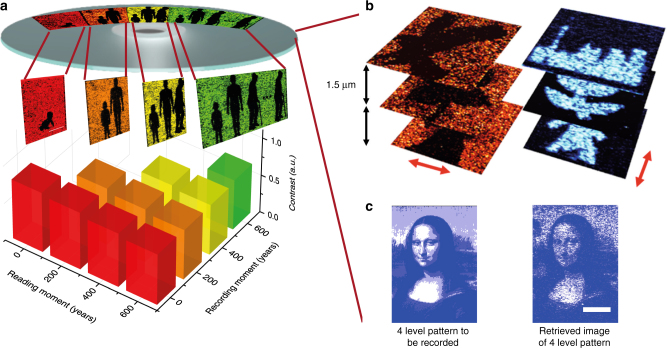
Century-long unchanged baseline in recording and reading processes of optical long data memory. **a** Contrast of the fluorescent images of the patterns at different recording and reading moments. Inset: Fluorescent images of the recorded patterns after aging over 0, 200, 400, and 600 years. Scale bar: 10 µm. **b** Multiplexed optical data memory with two polarization states in three layers spaced by 1.5 μm. The red arrow indicate the polarization direction of the writing laser beam. **c** Four-level optical data memory pattern in nanoplasmonic hybrid glass composites. Scale bar: 10 µm

To test the feasibility of optical data memory in the new nanoplasmonic hybrid glass composites, we used our home-built confocal setup to record and read patterns in the sample illuminated with a femtosecond laser beam. The photoreshaping of the nanorods in the new host matrix with enhanced Young’s modulus is further confirmed by studying the scattering spectrum of the single nanorod (Supplementary Note 4). In addition to a long lifespan, the enhanced Young’s modulus of the hybrid glass composites enables the precise control of the multi-length shape transition of nanorods for multi-level optical data memory. In contrast to the complete shape transition of gold nanorods from a rod to a sphere, where single gold nanorods serve as binary information carriers, the enhanced mechanical strength or elastic energy of the matrix allows for a multi-level optical data memory with long lifespan over 650 years by precisely controlling the stepwise shape transition with multi-length and, hence, the fluorescence strength of the recorded bits (Supplementary Note 3). The size of the recorded bits decreases when the Young’s moduli in the samples increase from 0.05 to 1.57 GPa with an enhanced melting threshold of nanorods. It should be noted that micro-explosion occurs in samples with Young’s moduli exceeding 1.57 GPa, which degrades the capacity owing to the increased size of single bits. The lateral and axial separations reach minimum when the Young’s modulus is increased to 1.57 GPa. (Fig. 3a) The equivalent capacity in the nanoplasmonic hybrid glass composites maximizes at 10 TB per disk at an optimized inorganic component ratio (Fig. 3b). The enhanced lifespans and increased capacity in a single nanoplasmonic hybrid glass disk open the potential for optical long data memory. As one of the most important aspect of long data memory, continuously recording and reading information in a single optical disk without changing the baseline is a necessary feature. To this purpose, events occurring at year 0 (red), year 200 (orange), year 400 (yellow), and year 600 (green) can be distinctively recorded at different positions in the same disk through the aging experiment, as shown in Fig. 4a. Here the recording moment and the reading moment indicate the time moment at which data are recorded and read the data after aging. To analyze the long data over such a long period of time, the recorded data should be read at any reading moment without changing the baseline. The data recorded in different recording moments are read at the same reading moment to confirm this requirement. The contrast of the retrieved image is measured to confirm the baseline. The contrast (*C*) of the pattern is calculated by

2 $$C = (I_{\max } - I_{\min })/(I_{\max } + I_{\min })$$

where *I*_max_ and *I*_min_ are the maximum and minimum value of fluorescent intensity of recorded patterns. After accelerated aging for 600 years, the contrast maintains a high level close to that read out immediately after recording. The contrast of the patterns at different recording moment shows the same baseline at the same reading moment. The data error is studied by calculate the correlation coefficient between the retrieved recorded image and the original image (Supplementary Note 4). The correlation coefficient is close to unity suggesting the images are spatially correlated with original data with low data error after aging of 600 years. The retrieved binary recorded image is still highly correlated to the original image even after aging of over 1000 years. This is of great importance to the active access of the long data at any reading moment without the degradation of the baseline. In addition, multiplexed optical data memory can be demonstrated by selective photoreshaping of gold nanorods with different lengths and orientations (see Fig. 4b and Supplementary Note 5). Figure 4c presents a four-level optical data memory pattern recorded with different power levels in a sample with a Young’s modulus of 1.57 GPa. Thus, the capacity can also be drastically enhanced in nanoplasmonic hybrid glass composites to store the extensive contents of long data. The capacity of 10 TB per disk can be achieved based on the polarization multiplexing and 4-level optical memory with lateral and axial separations between recorded bits are 650 nm and 1.5 µm, respectively.

## Discussion

The synthesis of the nanoplasmonic hybrid glass composite is based on a sol–gel process, which is compatible with the spin-coating method, paving a way to the low-cost large-scale mass-production of the new optical disks. This work can be the building blocks for the future of optical long data centers over centuries, unlocking the potential of the understanding of the long processes in astronomy, geology, biology, and history. It also opens new opportunities for high-reliability optical data memory that could survive in extreme conditions, such as high temperature and high pressure.

## Methods

### Material synthesis

Gold nanorods with an average aspect ratio of 2.7 and a diameter of 10 nm were prepared using wet chemical synthesis^29,30^. The nanorods were mixed with a hybrid sol–gel solution with organic and inorganic materials. Then the mixture was drop-casted on cover glasses. Thereafter, the cover glasses were placed in an oven at 313 K for a week (Supplementary Note 1).

### Nano-indentation test

The Young’s modulus of the samples was determined by nano-indentation (Hysitron Performech Ti750 Ubi Nanoindenter). Nine indentations arranged as a matrix of 3 × 3 (distance between two indentations is 3 µm) were tested for every sample. The loading force increased from 0 to 800 µN in 5 s and unloaded from 800 to 0 µN in 5 second after keeping at 800 µN for 10 s. The values of Young’s modulus were obtained by fitting the loading–unloading curves.

### Recording and reading of data

The recording and reading of the data were conducted in the same home-built confocal microscope with a femtosecond laser at a wavelength of 820 nm with a repetition rate of 80 MHz (SpectraPhysics, Maitai). The laser beam was focused onto the sample through an oil objective with a numerical aperture of 1.4 (Olympus). The storage pattern was recorded by raster scanning the sample with a piezo stage (PI corporate). The exposure time of each data points is 25 ms. The reading of the pattern was conducted by detecting the fluorescent signal of gold nanoparticles by a photomultiplier tube (Hamamatsu) with a 750 nm short pass filter with raster scanning of samples.

### Aging experiment

The aging experiment was conducted by heating the nanoplasmonic hybrid glass composites at 453 K in the oven. According to the measured lifespan of the shape of gold nanorods in Supplementary Note 3 in supplementary information, heating a sample at 453 K for three hours was equivalent to age the sample for 600 years at room temperature.

### Data availability

All data used to obtain the conclusions in this paper are available in the paper and/or the Supplementary Information. Other data may be requested from the authors.

## Electronic supplementary material

Supplementary Information(PDF 1235 kb)
