# Supplementary material for: High-capacity optical long data memory based on enhanced Young’s modulus in nanoplasmonic hybrid glass composites
Source: Nat Commun. 2018 Mar 22;9:1183. doi: 10.1038/s41467-018-03589-y (PMC5864957; doi:10.1038/s41467-018-03589-y)
Supplement: Supplementary file 1 — Supplementary Information(PDF 1235 kb) [file 41467_2018_3589_MOESM1_ESM.pdf]

Supplementary Information

High-capacity optical long data memory based on enhanced Young's modulus in nanoplasmonic hybrid glass composites

Zhang *et al*.

Supplementary Note 1. Sample preparation

The preparation process of the nanoplasmonic hybrid glass composites is shown in Supplementary Figure 1. Gold nanorods with an average aspect ratio of 2.7 and a diameter of 10 nm were prepared using wet chemical synthesis[1,2]. Nanoplasmonic hybrid glass composites were prepared by a sol–gel process. The regents include Tetramethoxysilane (TMOS), Poly(Ethylene Glycol) (PEG) (Mw = 400), Poly(ethylene glycol)-block-poly(propylene glycol)-block-poly(ethylene glycol) (P123) (Mw = 5800) and Hydrochloric acid (HCl) from Sigma-Aldrich. PEG was dissolved in water first, and then a P123 solution (20 wt% P123 in water) and TMOS were added. After being stirred at room temperature for 10 min, HCl solution (10 wt% Hydrochloric acid (32 wt% HCl in water) in water) was introduced. The solution was kept stirring unsealed at room temperature. Nanorods solutions (O.D. 180) were added into the hybrid sol, and then drop-casted on cover glasses. Thereafter, the cover glasses were placed in an oven at 313K for a week.

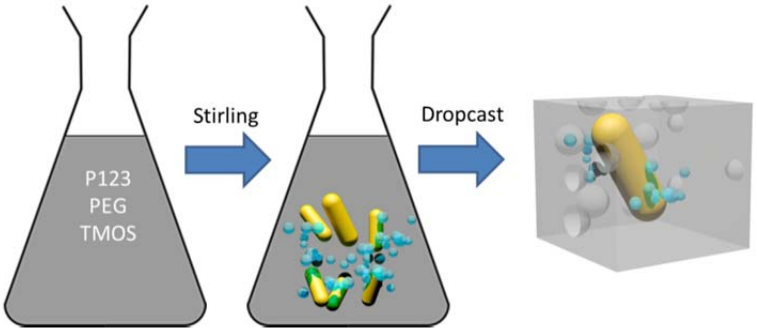

Supplementary Figure 1| Schematic of fabrication of nanoplasmonic hybrid glass composites. Fabrication process of the nanoplasmonic hybrid glass composites.

Supplementary Note 2. Theoretical investigation of the influence of the Young's modulus of the host matrix to the gold nanorods

The schematic of the expansion of the host matrix after the melting of the gold nanorods is shown in Supplementary Figure 2a. We assume that the gold nanorods change into gold nanospheres after the melting process. The radius and length of the nanorods is r_1 and l respectively. The radius of the gold nanospheres (r_2) after melting can be calculated by $r_2 = \sqrt[3]{\frac{3}{4} r_1^2 l}$ when considering the volume of gold is constant. The elastic (strain) energy (E_{elastic}) of the shape change by considering the host matrix expansion in the radial direction (r axis) and the axial direction (z axis) can be calculated according to the strain energy density function (U) in cylindrical coordinate[3],

$$E_{elastic} = \int_V U dV = \int_V \frac{1}{2}(\sigma_r \varepsilon_r + \sigma_z \varepsilon_z) dV$$

$$\sigma_r = \frac{Y}{(\mu+1)(2\mu-1)}[(\mu-1)\varepsilon_r - \mu\varepsilon_z] \tag{1}$$

$$\sigma_z = \frac{Y}{(\mu+1)(2\mu-1)}[(\mu-1)\varepsilon_z - \mu\varepsilon_r]$$

where σ_{r} and σ_{z} are stress in radial and axial directions, respectively, ε_{r} and ε_{z} are strain in radial and axial directions, respectively. Y is the Young's modulus of the host materials and μ is the Poisson's ratio. The ε_{r} and ε_{z} can be calculated based on the shape change of the host matrix from a cylinder with sphere heads (length: l, radius: r_1) into a spheroid (semi principal axis: $l/2$ and r_2). E_{elastic} can be calculated by numerical integration over the volume of the host matrix (V).

To further study the mechanical property of the nanoplasmonic hybrid glass composites, the Poisson's ratio of the hybrid glass composites with different inorganic percentages was tested by measuring the expansion perpendicular to the direction of compressing the bulk nanoplasmonic hybrid glass composites in a cylindrical shape (Diameter: 2 cm; Height: 3 cm) with a vernier scale.

Based on the experimental results of the Young's modulus in Fig. 2 in the main text and the Poisson's ratio in Supplementary Figure 2b, the elastic energy of the expansion of hybrid glass composites after the melting of gold nanorods is calculated based on equation (1) (Supplementary Figure 2c). The elastic energy is one order of magnitude higher than the melting energy of gold nanorods in air calculated from theory[4] when the percentage of the inorganic components is higher than 40 %. A melting threshold and activation energy of the reshaping of gold nanorods to nanospheres in hybrid glass composites, higher than those in air, can be expected to suppress the elastic energy of the host matrix.

As shown in the energy barrier model of the data storage with gold nanorods in the inset of Fig. 2a in the main text, the data is stored in the energy minima of the system between the nanorods and nanospheres. According to the theoretical simulation, the gold nanorods have a free energy higher than that of the spheres. Thus the lifespan of the optical data memory in nanoplasmonic hybrid glass composites mainly depends on the lifespan of shape of nanorods. Activation energy proportional to the melting threshold of nanorods is required to change the shape from a rod to a sphere. At the temperature of 0 K there are no thermal fluctuations and the shape will stay in a rod indefinitely, but at elevated temperatures the probability that the shape jump from a rod to a sphere increases according to the Arrhenius law. As a consequence of the enhanced Young's modulus of the host matrix components, additional activation energy is essential for the shape transition of the nanorods to overcome the elastic energy associated to the enhanced Young's modulus during the expansion of the host matrix. An enhancement of activation energy reduces the probability of jumping between different shapes due to the thermal fluctuation, thus enhancing the lifespan of the shape of the nanorods.

To gain a better insight into the temperature rise of the surrounding matrix on the gold nanorods excited by the femtosecond laser beam at a high repetition rate, the rising of the focal temperature in the matrix is modeled by considering gold nanorods as the heat sources. The local temperature change during the writing process is estimated based on the heat

conduction equation[5]. The spatial and temporal dependence of the temperature change within the focal region in the sample is given by

$$\nabla T(r,t)=\sum_{n=1}^{m}\frac{F(r_0)a}{8k(\frac{k}{\rho c_p})^{\frac{1}{2}}(\pi nt)^{\frac{3}{2}}}\exp(-\frac{(r-r_0)^2}{4\frac{k}{\rho c_p}nt}) \tag{2}$$

Here $F(r_0)$ is the energy-density distribution given by the objective, a is the absorption cross-section of gold nanorods (~6×10^{-12} cm^2), ρ is the density of the hybrid glass composites with different inorganic percentages measured in experiment (1200 kg cm^{-3} (0 %); 1800 kg cm^{-3} (40 %); 2000 kg cm^{-3} (60%); 2400 kg cm^{-3} (80%); 2700 kg cm^{-3} (100%)), c_p is the heat capacity of the hybrid glass composites with different inorganic percentages measured by differential scanning calorimetry (DSC 4000, Perkin Elmar) (1700 J kg^{-1} K^{-1} (0 %); 1300 J kg^{-1} K^{-1} (40 %); 1000 J kg^{-1} K^{-1} (60 %); 850 J kg^{-1} K^{-1} (80 %); 700 J kg^{-1} K^{-1} (100 %)), k is the thermal conductivity of the hybrid glass composites with different inorganic percentages measured by differential scanning calorimetry (DSC 4000, Perkin Elmar) (0.20 W m^{-1} K^{-1} (0 %); 0.65 W m^{-1} K^{-1} (40 %); 0.85 W m^{-1} K^{-1} (60 %); 1.10 W m^{-1} K^{-1} (80 %); 1.30 W m^{-1} K^{-1} (100 %)), t is the time interval between two successive pulses (12.5 ns for the repetition rate of 80 MHz), n is the number of pulses, r-r_0 is the relative distance from the gold nanorods. The nanorods are modeled as a homogeneous distribution in the matrix with a particle separation of 80 nm to simulate the experimental condition. The initial temperature of 293 K is assumed. The average focal temperature is obtained by superposing the solutions of every gold nanorod inside the focus. Supplementary Figure 2d shows the calculated focal temperature of the nanoplasmonic hybrid glass composites excited by a femtosecond laser at 820 nm with an average power of 8 mW and an exposure time of 25 ms. This result shows that the temperature at the matrix during the writing process is lower than 333 K (60 ^{0}C) at a high inorganic level of the hybrid glass composites, which is far below the melting point of the matrix. Therefore, the melting of the matrix during in the calculation of the elastic energy is not considered.

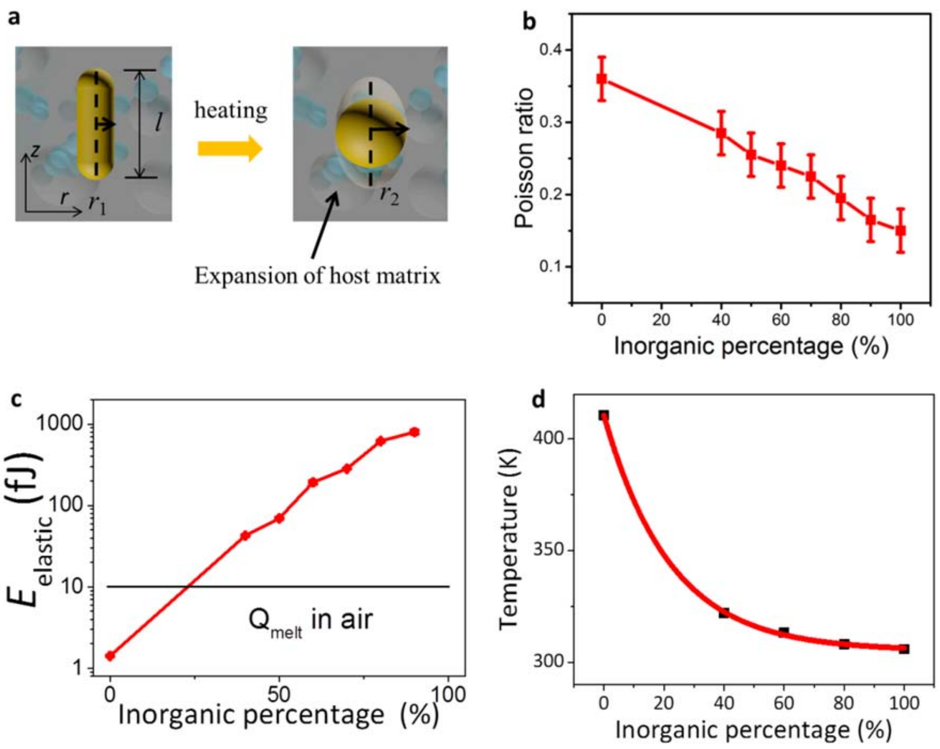

Supplementary Figure 2| Theoretical analysis of the influence of the host matrix to the thermal stability of the shape of gold nanorods. a. Schematic of the expansion of the host matrix after the melting of the nanorods. **b.** Experimental result of Poisson's ratio. Standard deviations are represented by error bars. **c.** Calculated elastic energy of the host matrix expansion after the melting of the nanorods. **d.** Simulation result of the average temperature around the focal spot.

Supplementary Note 3. Accelerated aging experiment in nanoplasmonic hybrid glass composites

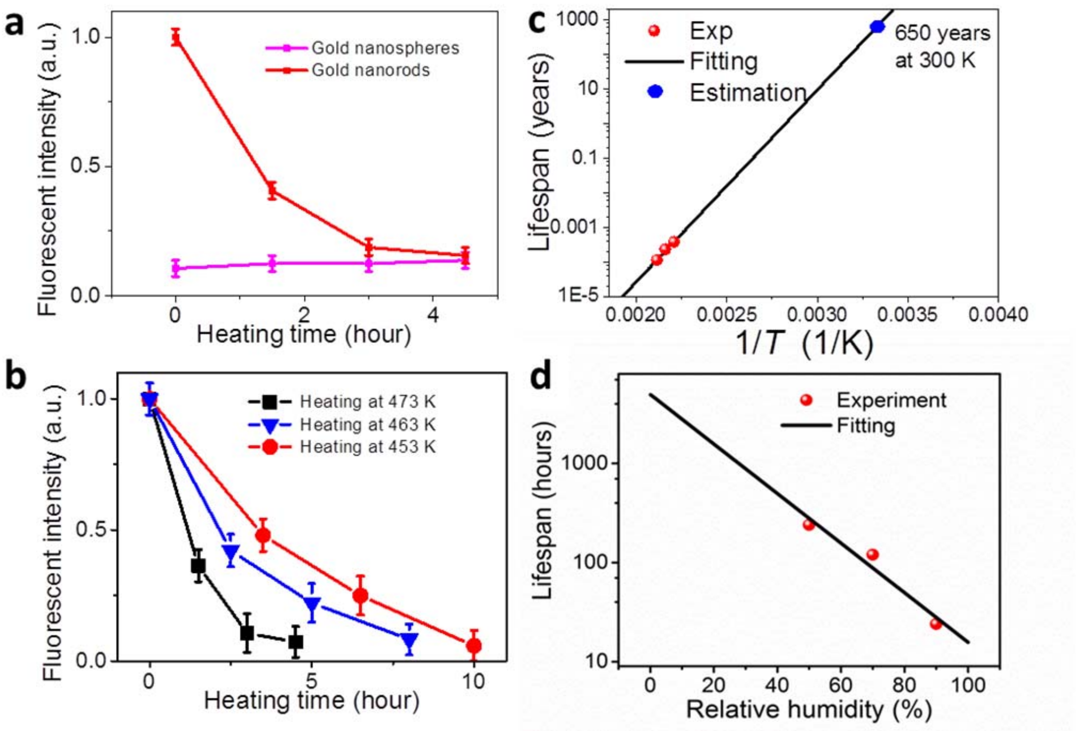

Supplementary Figure 3| Accelerated aging experiment of gold nanorods in nanoplasmonic hybrid glass composites. a. Fluorescent intensity of the gold nanorods and nanospheres after heating. Standard deviations are represented by error bars. **b.** Fluorescent intensity of gold nanorods after heating at different temperatures. Standard deviations are represented by error bars. **c.** Lifespan estimation of the shape of gold nanorods in nanoplasmonic hybrid glass composites at an elevated temperature. **d.** Lifespan of the shape of gold nanorods in nanoplasmonic hybrid glass composites at different humidity conditions at 358 K.

The accelerated aging experiment was performed by heating the sample with high density of nanorods in an oven at an elevated temperature. After heating the sample with 40% of inorganic components, the fluorescent intensity of the gold nanospheres generated from nanorods by photoreshaping with a femtosecond laser beam did not change after aging (Supplementary Figure 3a). The fluorescent intensity of the gold nanorods in the hybrid glass composites decreased. As shown in Supplementary Figure 3b, the time constant of the lifespan (L) of the shape of gold nanorods in different heating temperatures were determined when the intensity of the fluorescent signal of nanorods decreased to half of the intensity before heating. To estimate the lifespan at room temperature, we have fit the acquired

lifespans at high temperature with equation (1) in the main text, by transferring equation (1) in the main text into linear relation,

$$\ln(L) = E_a / k_B T + \ln(A) \quad (3)$$

By fitting the lifespan at different temperature in Supplementary Figure 3c with equation (3), the activation energy (E_a) is 7.67×10^{-20} J and the pre-exponential factor (A) is 2.06×10^{-16}. Based on the fitting parameters, the estimated lifespan at room temperature (300 K) is 650 years (Supplementary Figure 3c).

To study the influence of the humidity on the lifespan of the glass composite, we performed accelerated aging experiment in the humidity control box at different humidity levels at a temperature of 358 K as shown in Supplementary Figure 3d. The lifespan decreases as the relative humidity (RH) level increases. The effect of *RH* on lifespan was modeled from the Eyring equation based on the modification of the Arrhenius equation[6]:

$$L = A\exp(E_a / k_B T + B \cdot RH) \quad (4)$$

where B is a pre-exponential factor for relative humidity. The equation (3) can be transformed into linear relation,

$$\ln(L) = E_a / k_B T + \ln(A) + B \cdot RH \quad (5)$$

By fitting experiment al results in Fig. S3d, the pre-exponential factor B is -2.5×10^{-2}. By implementing the E_a and A values from the elevated temperature aging experiment in Supplementary Figure 3c in equation (3), the estimated lifespan at the room temperature (300 K) and a relative humidity of 5 % is 480 years.

To further confirm the mechanism of the reduced fluorescent intensity of nanorods in the accelerated aging experiment, the white light scattering spectra of the optical data memory media with high density of nanorods were measured after heating in the oven. The scattering spectra of the sample before and after aging at 393 K for 1 hour are shown in Supplementary Figure 4. A blue shift of the scattering peak of the nanorods indicates a reduction of the aspect ratio. The reduction of the fluorescent signal is originated from the

shape change of the gold nanorods. An increase of the thermal stability of gold nanorods has been confirmed after the enhancement of the Young's modulus of the host matrix.

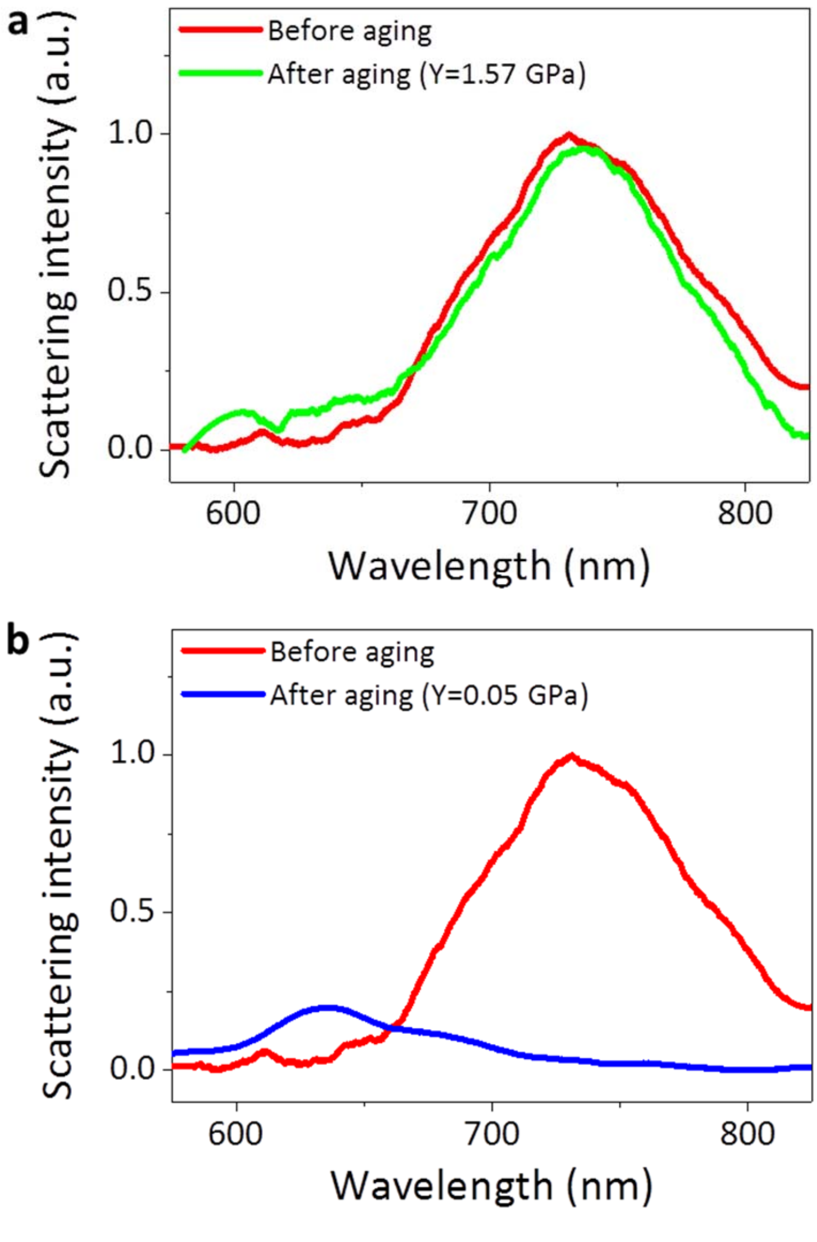

Supplementary Figure 4| White light scattering spectra of nanorods. Scattering spectra of gold nanorods in the nanoplasmonic hybrid glass composites with Young's modulus (Y) of 1.57 GPa (**a**) and 0.05 GPa (**b**) after heating at 393 K for 1 hour.

Supplementary Note 4. Study of the photoreshaping of gold nanorods in the hybrid glass composites

To confirm the shape transformation of a single nanorod in hybrid glass composites with high Young's modulus during the femtosecond laser irradiation for optical data memory, we performed the white light scattering experiment. The schematic drawing of the experimental setup is shown in Supplementary Figure 5a. The output from a halogen white light source was spatially filtered and collimated. The central of the beam was blocked by a circular obstruction disk (ε = 0.8) to form an annular illumination and focused onto the sample through an oil immersion objective (Olympus, NA = 1.4). The back scattering light was collected by the same objective. A diaphragm with a diameter smaller than the size of the circular obstruction disk was used in the scattering light to enhance the contrast. Then the beam was directed to a photomultiplier tube (Hamamatsu) and a spectrometer (Acton Instruments). A femtosecond laser beam at a wavelength of 820 nm and a repetition rate of 80 MHz (Spectra Physics, Maitai) was aligned into the setup for laser melting and a half wave plate was used to control the polarization to match the orientation of nanorods.

Supplementary Figure 5b shows the white light scattering spectra of the a single nanorod in nanoplasmonic hybrid glass composites (40% of inorganic components, Y=1.57 GPa) before and after the irradiation of the femtosecond laser at 225 μW for 25 ms. A scattering peak of the gold nanorod shifted to a shorter wavelength after the irradiation of the femtosecond laser. The aspect ratio of the nanorod can be projected from the resonant peak position[7]. The shape transition at different melting laser power levels is shown in Supplementary Figure 5c. The melting threshold of a single nanorod in nanoplasmonic hybrid glass composites increases as the Young's modulus enhances.

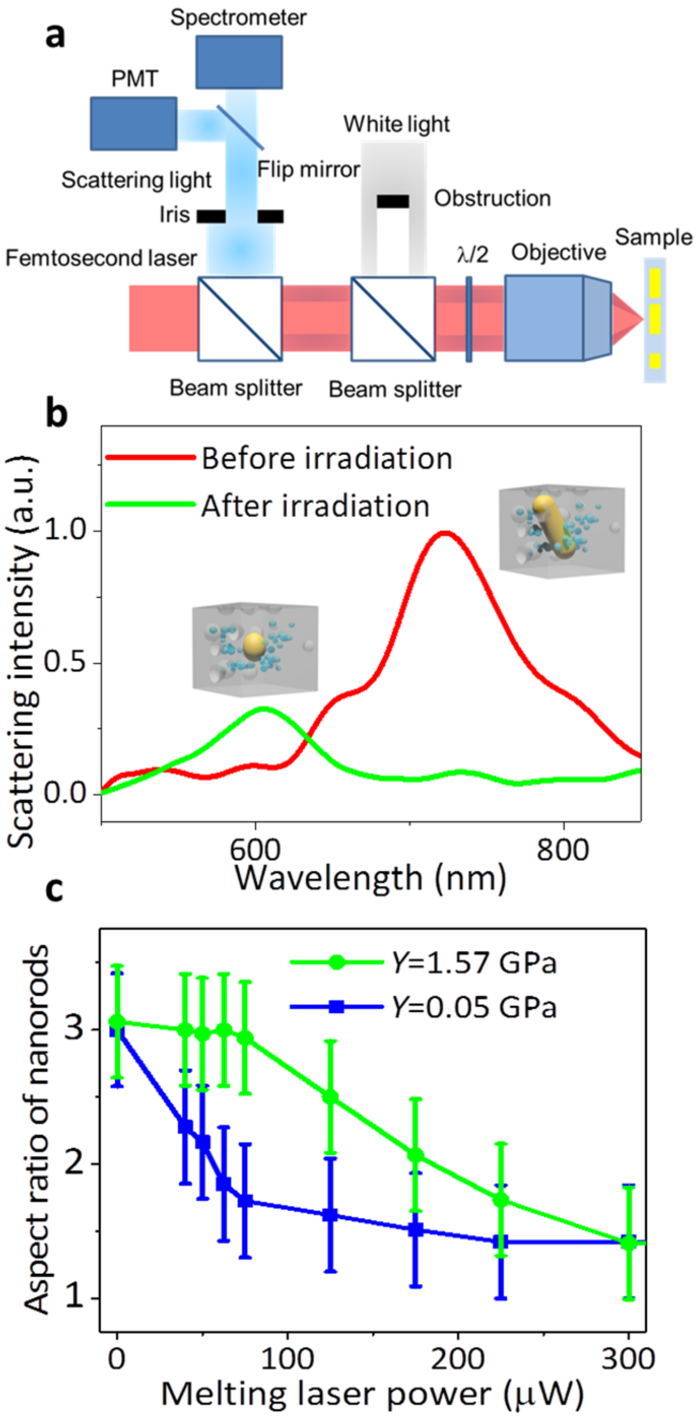

Supplementary Figure 5| White light scattering spectra of single gold nanorods. a. Schematic drawing of the setup for white light scattering spectroscopy and laser melting of single nanorods. **b.** Scattering spectra of single nanorods. **c.** Aspect ratio of single gold nanorods at different melting laser power levels. Standard deviations are represented by error bars.

To analyze the data error during the aging process, we analyzed the correlation and data errors ratio between the recorded two-dimensional images after the aging experiment at different reading moments with the original images. As shown in the Supplementary Figure

6b, the two-level recorded images are retrieved by binarizing the experimental data with command “imbinarize” in Matlab and then compared with the original two-level image with 73×73 pixels. The correlation between the recorded images and original images can be defined by the Pearson product-moment correlation coefficient (C) between two matrices[8] as follows:

$$C(X,Y)=\frac{\sum_m\sum_n(X_{mn}-\overline{X})(Y_{mn}-\overline{Y})}{\sqrt{\sum_m\sum_n(X_{mn}-\overline{X})^2}\sqrt{\sum_m\sum_n(Y_{mn}-\overline{Y})^2}} \quad (6)$$

where X_{mn} and Y_{mn} is the intensity of data bits in the recorded and original images, $\overline{X}$ and $\overline{Y}$ is the average intensity of all the data bits in the recorded and original images. The correlation coefficient of unity corresponds to two identical images while a value of zero corresponds to no overlap between the two images. The data error ratio was calculated by the error bits divided by total number of pixels (73×73=5329). As shown in the Supplementary Figure 6b, the correlation coefficient is close to unity suggesting the images are spatially correlated with original data with low data error after aging of 600 years. The retrieved binary recorded image is still highly correlated to the original image even after aging of over 1000 years.

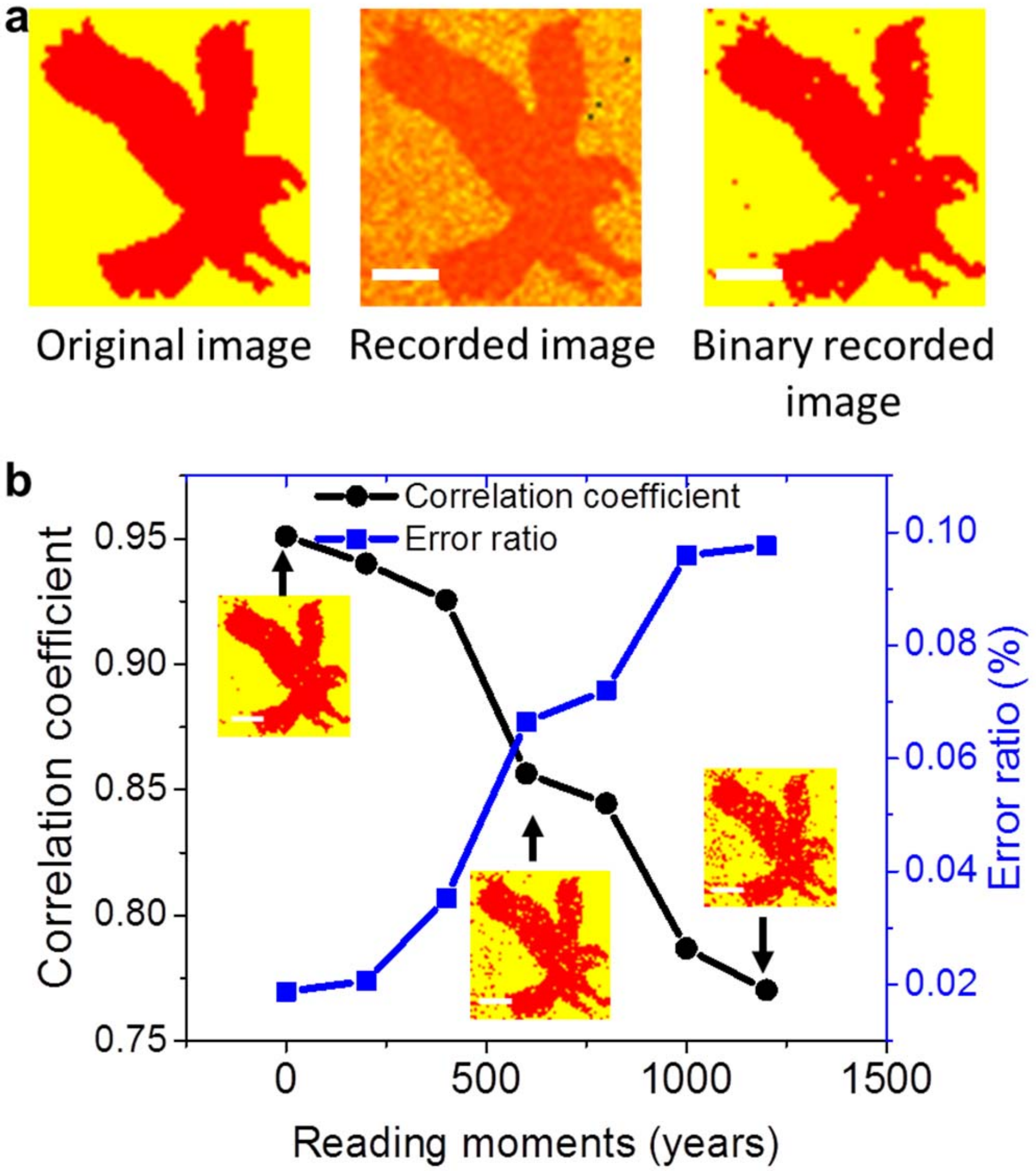

Supplementary Figure 6| Study of the data error of optical data memory in the nanoplasmonic hybrid glass composites at different reading moments. a. The original image, recorded image obtained by detecting the fluorescent signal of all the information bits and the binary recorded image retrieved by binarizing the recorded image. Scare bar: 10 µm. **b.** Correlation coefficient and data error ratio of binary recorded image at different reading moments of nanoplasmonic hybrid glass composites after aging experiment at 453 K. Inset: binary recorded images at different reading moments. Scale bar: 10 µm.

To confirm the influence of the matrix to the melting of gold nanorods in the optical data memory, we performed experiments on the recording power of the sample with a high concentration of nanorods. Supplementary Figure 7 shows the fluorescent intensity of the nanoplasmonic hybrid glass composites with a Young's modulus of 1.57 GPa after the irradiation with a femtosecond laser beam at a wavelength of 820 nm with different power

levels. For comparison, a nanoplasmonic hybrid glass composite with a Young's modulus of 0.05 GPa was also studied. The nanorods in low Young's modulus host matrix changed into nanospheres at a power level much lower than that in hybrid glass composites with high Young's modulus.

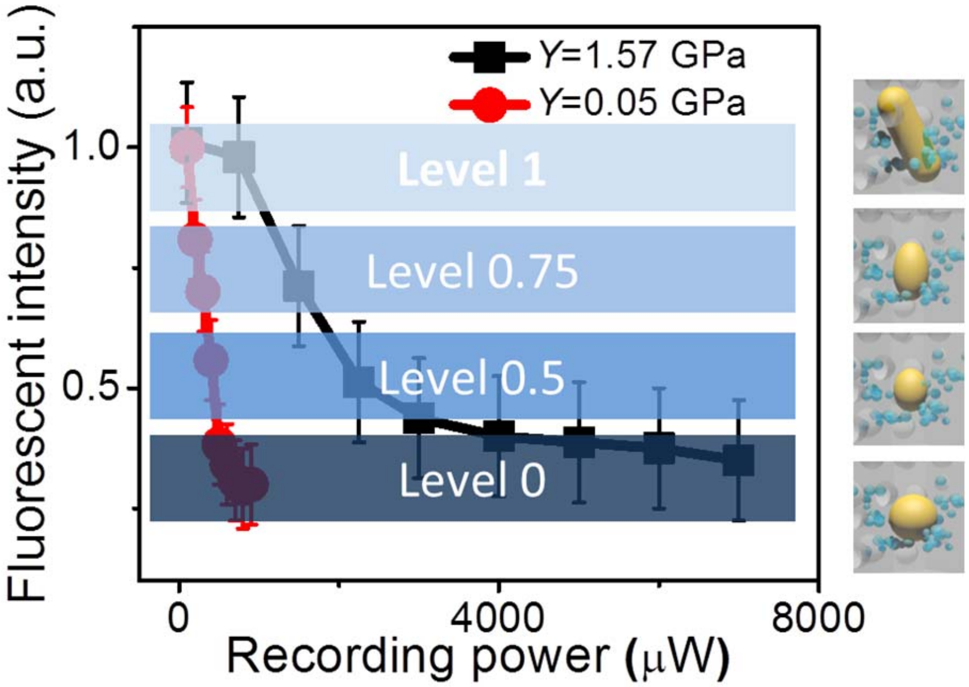

Supplementary Figure 7| Study of the melting threshold of nanorods in the nanoplasmonic hybrid glass composites. Fluorescent intensity of gold nanorods after recording at different laser power levels. Standard deviations are represented by error bars.

Supplementary Note 5. Demonstration of multiplexed optical data memory

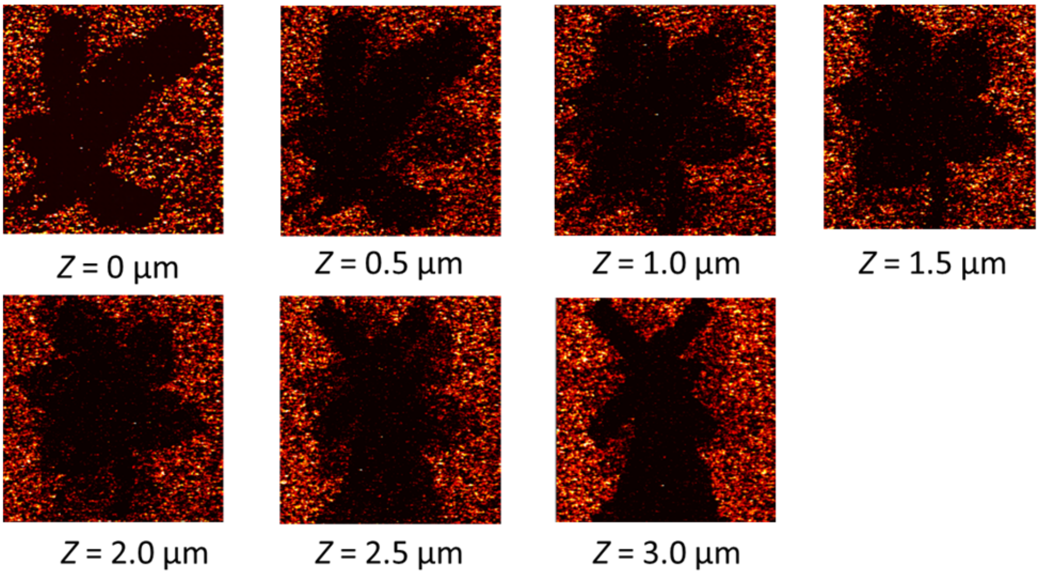

Supplementary Figure 8| Multiplexed optical data memory. Reading of three-layer optical data memory in different depths.

Six images were patterned at a wavelength of 820 nm in three layers in the same area at different depths spaced by 1.5 μm (Fig. 4 in the main text). Two patterns were recorded in two orthogonal polarization states at each layer. To demonstrate the separation of our multi-layer optical data memory, the patterns were read by scanning the sample in different depths (Supplementary Figure 8).

Supplementary References

1 Nikoobakht, B. & El-Sayed, M. A. Preparation and growth mechanism of gold nanorods (NRs) using seed-mediated growth method. *Chem Mater* **15**, 1957-1962, (2003).

2 Zijlstra, P., Bullen, C., Chon, J. W. M. & Gu, M. High-temperature seedless synthesis of gold nanorods. *J Phys Chem B* **110**, 19315-19318 (2006).

3 Murakami, Y. *Theory of elasticity and stress concentration*. (John Wiley & Sons, 2016).

4 Bohren, C. F. & Huffman, D. R. *Absorption and scattering of light by small particles*. (Wiley, 1983).

5 Ullah, M. A. *et al.* Low energy-density recording with a high-repetition-rate laser beam in gold-nanorod-embedded discs. *Opt Express* **20**, 24516-24523 (2012).

6 Glasstone, S., Laidler, K. J. & Eyring, H. The theory of rate processes; the kinetics of chemical reactions, viscosity, diffusion and electrochemical phenomena. (McGraw-Hill Book Company, 1941).

7 Zijlstra, P., Chon, J. W. M. & Gu, M. White light scattering spectroscopy and electron microscopy of laser induced melting in single gold nanorods. *Phys Chem Chem Phys* **11**, 5915-5921, (2009).

8 Rosenhead, J. V. The advanced theory of statistics, Vol 2, Inference and relationship - Kendall,Mg, Stuart,A. *Oper Res Quart* **14**, 97-98, (1963).
